# Tooth Loss, Denture Use, and Risk of Malnutrition in Older Adults in Poland: Evidence from the National PolSenior2 Study

**DOI:** 10.3390/nu18061010

**Published:** 2026-03-23

**Authors:** Wojciech Dąbrowski, Aleksandra Kaluźniak-Szymanowska, Kacper Jagiełło, Łukasz Wierucki, Renata Górska, Hanna Kujawska-Danecka, Katarzyna Wieczorowska-Tobis

**Affiliations:** 1 Department of Dental Prosthetics, Faculty of Medicine, Medical University of Gdańsk, 80-210 Gdańsk, Poland; 2 Geriatric Unit, Department of Palliative Medicine, Poznań University of Medical Sciences, 61-701 Poznań, Poland; akaluzniak-szymanowska@ump.edu.pl (A.K.-S.); kwt@tobis.pl (K.W.-T.); 3 Division of Preventive Medicine and Education, Faculty of Medicine, Medical University of Gdańsk, 80-210 Gdańsk, Poland; kacper.jagiello@gumed.edu.pl (K.J.); lukasz.wierucki@gumed.edu.pl (Ł.W.); 4 Department of Periodontology and Oral Diseases, Medical University of Warsaw, 02-091 Warsaw, Poland; renata.gorska@wum.edu.pl; 5 Department of Rheumatology, Clinical Immunology, Geriatrics and Internal Medicine, Medical University of Gdańsk, 80-210 Gdańsk, Poland; hanna.kujawska@gumed.edu.pl; 6 Department of Human Nutrition and Dietetics, Poznań University of Life Sciences, 60-637 Poznań, Poland

**Keywords:** malnutrition, mini nutritional assessment–short form (MNA-SF), older adults, dentition status, edentulism, denture use, impaired nutritional status

## Abstract

**Background**: Malnutrition and its risk are prevalent in older adults and contribute to frailty, morbidity, and mortality. Poor oral health—particularly tooth loss and inadequate prosthetic rehabilitation—may impair chewing, limit dietary variety, and accelerate nutritional decline. We investigated associations between dentition status, denture use, and nutritional status in a nationally representative sample of Polish older adults. **Methods**: We analyzed data from 5214 participants aged ≥60 years from the nationwide cross-sectional PolSenior2 study. Dentition status was classified as functional dentition (≥20 teeth), partial dentition (1–19 teeth), or edentulism (0 teeth). Nutritional status was assessed using the Mini Nutritional Assessment—Short Form (MNA-SF); impaired nutritional status was defined as MNA-SF <12 (malnourished or at risk). Additional indicators included hypoalbuminemia (<35 g/L) and small calf circumference (<31 cm). Associations were tested using chi-square and multivariable logistic regression adjusted for age, sex, education, and place of residence. **Results**: Functional dentition was present in 15.5%, partial dentition in 48.1%, and edentulism in 36.4% of participants. The proportion of edentulous individuals increased across worsening MNA-SF categories (26.2% in well-nourished, 41.8% in at risk, 46.9% in malnourished). In adjusted models, edentulism was associated with higher odds of impaired nutritional status compared with functional dentition (OR: 1.66; 95% CI: 1.32–2.10; *p* < 0.001), while partial dentition showed a non-significant trend (OR: 1.22; 95% CI: 0.98–1.52; *p* = 0.077). Among edentulous participants, denture use was more common in well-nourished individuals than in those with impaired nutritional status (93.0% vs. 77.2%), suggesting a possible association between active prosthetic rehabilitation and better nutritional status. **Conclusions**: In Polish older adults, tooth loss—particularly edentulism—is associated with poorer nutritional status. Screening for malnutrition risk may benefit from incorporating basic oral health and denture-use assessment while improved access to prosthetic rehabilitation may support nutritional resilience in ageing populations.

## 1. Introduction

Population ageing is associated with a growing burden of geriatric conditions, among which, malnutrition and deteriorating oral health play a particularly important role. Both conditions substantially affect physical functioning, the course of chronic diseases, and quality of life in older adults [1,2,3,4]. Malnutrition often remains undiagnosed until advanced stages despite its well-documented consequences, including sarcopenia, impaired immune response, delayed wound healing, functional decline, and increased mortality.

Adequate nutrition is a key determinant of healthy ageing and maintenance of physical and metabolic function across the lifespan. In older adults, insufficient dietary intake and inadequate nutrient availability may contribute to sarcopenia, functional decline, and increased vulnerability to chronic disease. In this context, nutritional adequacy and, when appropriate, targeted dietary supplementation play an important role in maintaining physical performance, metabolic balance, and overall health in ageing populations.

Global estimates indicate that nearly one in five adults aged ≥65 years is affected by malnutrition or is at risk, with prevalence increasing markedly in the oldest age groups and among individuals with multimorbidity or functional limitations [5,6].

In recent years, increasing attention has been directed toward potentially modifiable determinants of nutritional status in later life. Among these, oral health has emerged as an important but frequently overlooked factor. Tooth loss, inadequate prosthetic rehabilitation, and impaired oral function may directly compromise food intake by limiting chewing efficiency, reducing dietary variety, and discouraging the consumption of nutritionally dense foods, including fresh fruits, vegetables, nuts, whole-grain products, and protein-rich foods [7,8]. Consequently, older adults with limited dentition are more likely to follow softer, energy-dense but nutrient-poor diets, which may contribute to protein–energy malnutrition and micronutrient deficiencies [9,10].

Beyond mechanical aspects of mastication, oral health influences nutrition through additional biological and behavioral pathways. Xerostomia—highly prevalent in older adults due to polypharmacy, salivary gland dysfunction, or chronic diseases—impairs bolus formation and swallowing, reduces comfort during meals, and further restricts food choice and intake [9,11]. Psychosocial factors also play a significant role. Edentulism and oral discomfort may negatively affect self-esteem and social participation, leading to avoidance of eating in social settings, suppressed appetite, and reduced meal frequency. Functional limitations, difficulties in meal preparation, cognitive impairment, and social isolation may further exacerbate inadequate nutritional intake and create a vicious cycle linking oral dysfunction and malnutrition [12,13].

Evidence from observational studies and systematic reviews consistently shows that older adults with fewer teeth, poor subjective oral health, chewing difficulties, or poorly fitting dentures are more likely to have suboptimal diets and a higher risk of malnutrition [7,8,9,10]. Conversely, appropriate prosthetic rehabilitation may improve chewing efficiency, facilitate a more balanced diet, and potentially mitigate nutritional decline associated with tooth loss [14,15]. However, most available evidence originates from Western Europe and East Asia, and large population-based studies integrating objective measures of dentition, denture use, and standardized nutritional screening tools remain scarce—particularly in Central and Eastern Europe, where the prevalence of edentulism and unmet prosthetic needs remains high.

Recent analyses of the PolSenior and PolSenior2 cohorts have demonstrated a significant improvement in dental status among Polish older adults over the last decade, including a decline in edentulism and an increase in functional dentition, although substantial sociodemographic disparities persist [16]. In parallel, national analyses have shown a significant reduction in unmet denture needs among Polish seniors between 2009 and 2019; however, persistent inequalities in prosthetic coverage related to sex, place of residence, education, and financial status remain [17]. Nonetheless, the nutritional implications of these oral health changes have not been comprehensively evaluated. Poland represents a relevant setting for investigating these relationships due to its rapidly ageing population and persistent inequalities in oral health and access to prosthetic care among older adults. Importantly, few studies have simultaneously examined dentition status, denture use, and nutritional outcomes using validated screening instruments such as the Mini Nutritional Assessment—Short Form (MNA-SF), alongside anthropometric and laboratory indicators related to frailty and nutritional risk.

Therefore, the aim of the present study was to investigate the association between dentition status, denture use, and nutritional status in a nationally representative sample of older adults in Poland. We hypothesized that tooth loss—particularly edentulism—would be associated with a higher risk of impaired nutritional status and that denture use among edentulous individuals may be associated with more favorable nutritional profiles.

## 2. Materials and Methods

### 2.1. Study Design and Population

This study is based on data from PolSenior2, a nationwide, population-based, cross-sectional survey conducted in Poland in 2018–2019 among adults aged 60 years and older. The PolSenior2 project included 5987 participants who completed the questionnaire-based part of the survey and underwent anthropometric and blood pressure measurements. Participants were selected using a three-stage stratified, clustered, and proportional random sampling procedure designed to obtain a sample representative of the older Polish population with respect to age, sex, and place of residence. To ensure sufficient statistical power in the oldest age groups, the sampling design included seven approximately equally sized age cohorts.

Data collection was performed during home visits by trained nurses and included face-to-face interviews, standardized geriatric scales and tests, anthropometric measurements, and collection of blood and urine samples. Detailed information on the design and methodology of the PolSenior2 study has been published elsewhere.

For the present analysis, participants were eligible if information on dentition status and at least one indicator of nutritional status was available, including Mini Nutritional Assessment–Short Form (MNA-SF) responses, serum albumin concentration, or calf circumference. Individuals with missing data for key variables required for the respective analyses were excluded. The final analytical sample consisted of 5214 older adults.

### 2.2. Ethical Considerations

The PolSenior2 study was conducted in accordance with the principles of the Declaration of Helsinki and applicable national regulations. All participants provided written informed consent prior to participation. The study protocol was approved by the Independent Bioethics Committee of the Medical University of Gdańsk (approval No. NKBBN/257/2017) [18].

### 2.3. Assessment of Dentition Status and Denture Use

Dentition status was determined based on the number of natural teeth present during clinical examination. Participants were classified into three categories:

- Functional dentition: ≥20 natural teeth;
- Partial dentition: 1–19 natural teeth;
- Edentulism: 0 natural teeth.

Information on prosthetic rehabilitation was collected separately. The presence and use of complete dentures in the maxilla and mandible were recorded. For analytical purposes, edentulous participants were classified as denture users or non-users, with the latter group including individuals who did not possess dentures or did not use them.

### 2.4. Assessment of Nutritional Status

Nutritional status was assessed using the Mini Nutritional Assessment—Short Form (MNA-SF), a validated screening tool widely used in older populations. Based on the total score, participants were categorized as:

- Malnourished (0–7 points);
- At risk of malnutrition (8–11 points);
- Well nourished (12–14 points).

For regression analyses, impaired nutritional status was defined as MNA-SF <12, encompassing both malnourished individuals and those at risk of malnutrition.

In addition to MNA-SF, two objective indicators related to nutritional status were analyzed:

- Hypoalbuminemia, defined as serum albumin concentration <35 g/L;
- Small calf circumference, defined as <31 cm, indicating increased risk of low muscle mass.

### 2.5. Statistical Analysis

Descriptive statistics were used to characterize the study population. Categorical variables were presented as percentages with 95% confidence intervals (95% CIs). Group comparisons were performed using the chi-square test.

Associations between dentition status, denture use, and nutritional status categories were analyzed using univariate methods. The relationship between denture use and nutritional status was examined separately in the subgroup of edentulous participants.

Multivariable logistic regression models were constructed to estimate odds ratios (ORs) and 95% confidence intervals for impaired nutritional status (MNA-SF < 12) according to dentition status. Functional dentition served as the reference category. The models were adjusted for potential confounders, including age, sex, education level, and place of residence. Age- and sex-stratified analyses were not performed; instead, these variables were included as covariates in the multivariable models.

A two-sided *p*-value <0.05 was considered statistically significant. Statistical analyses were performed using Statistica 13.3 (TIBCO Software Inc., Palo Alto, CA, USA) and R software (version 4.2.2).

Participants with missing key variables required for the primary analyses were excluded from the respective models. The analytical sample therefore included individuals with available data on dentition status and at least one nutritional indicator. Because the study has a cross-sectional design, causal relationships between dentition status and nutritional outcomes could not be inferred.

## 3. Results

### 3.1. Characteristics of the Study Population

The analysis included 5214 individuals aged ≥60 years, of whom, 51.6% were women. The largest age group comprised participants aged 60–69 years (34.0%), followed by those aged 70–79 years (33.1%) and 80–89 years (24.2%), while individuals aged ≥90 years accounted for 8.7% of the study population. Regarding place of residence, 36.2% of respondents lived in rural areas, 25.4% in towns with fewer than 50,000 inhabitants, 18.8% in cities with 50,000–200,000 inhabitants, and 19.7% in cities with more than 200,000 inhabitants. Functional dentition (≥20 teeth) was observed in 15.5% of participants, partial dentition (1–19 teeth) in 48.1%, and edentulism in 36.4%. Detailed sociodemographic characteristics of the study population are presented in Table 1.

### 3.2. Dentition Status and MNA-SF Categories

A clear gradient of worsening nutritional status with increasing tooth loss was observed. Among well-nourished individuals, edentulism was present in 26.2% (95% CI: 23.8–28.5), whereas functional dentition was identified in 23.6% (95% CI: 21.2–25.9). In participants at risk of malnutrition, the proportion of edentulous individuals increased to 41.8% (95% CI: 38.2–45.4), while the proportion with functional dentition decreased to 12.9% (95% CI: 9.5–16.2). The highest prevalence of edentulism was observed among malnourished individuals, reaching 46.9% (95% CI: 34.9–58.9), whereas functional dentition was rare in this group (9.3%; 95% CI: 0.9–17.7). The proportion of participants with partial dentition remained relatively stable across MNA-SF categories (50.3%, 45.3%, and 43.8%, respectively). Detailed results are shown in Table 2.

Overall, edentulism occurred nearly twice as often as functional dentition among malnourished individuals, indicating a strong association between tooth loss and poorer nutritional status.

### 3.3. Dentition Status in Relation to Albumin Concentration and Calf Circumference

Indicators related to frailty and nutritional risk were higher among edentulous participants. Among individuals with hypoalbuminemia (<35 g/L), edentulism was observed in 38.0% compared with 29.7% among those with albumin concentrations ≥35 g/L.

A similar pattern was noted for calf circumference. In participants with calf circumference <31 cm, edentulism was present in 44.1%, whereas in those with calf circumference ≥33 cm, the corresponding proportion was 28.3%. Detailed results are presented in Table 3.

### 3.4. Denture Use Among Edentulous Participants and Nutritional Status

The subgroup analysis included 1900 edentulous participants, of whom, 88.1% (*n* = 1673) reported denture use and 11.9% (*n* = 226) did not possess or did not use dentures. Among well-nourished edentulous individuals, 93.0% (95% CI: 90.9–95.1) used dentures, while 7.0% (95% CI: 4.9–9.1) did not. In participants at risk of malnutrition, denture use was reported by 84.1% (95% CI: 80.0–88.2), whereas 15.9% (95% CI: 11.8–20.0) were non-users. Among malnourished individuals, the respective proportions were 77.2% (95% CI: 67.1–87.2) and 22.8% (95% CI: 12.8–32.9). Detailed results are presented in Table 4.

The absence of active prosthetic rehabilitation became progressively more common with worsening nutritional status. In malnourished participants, lack of denture use occurred more than three times as often as in well-nourished individuals, suggesting that denture use may be associated with more favorable nutritional statuses.

### 3.5. Multivariable Analysis

In the multivariable logistic regression model, compared with participants with functional dentition, edentulism was associated with a significantly increased risk of impaired nutritional status (MNA-SF < 12) (OR: 1.66; 95% CI: 1.32–2.10; *p* < 0.001). Partial dentition showed a non-significant trend toward increased risk (OR: 1.22; 95% CI: 0.98–1.52; *p* = 0.077). The risk of impaired nutritional status increased markedly with age. Compared with participants aged 60–69 years, the odds ratios were 1.27 (95% CI: 1.07–1.51; *p* = 0.006) for those aged 70–79 years, 2.37 (95% CI: 1.97–2.84; *p* < 0.001) for those aged 80–89 years, and 5.19 (95% CI: 4.04–6.65; *p* < 0.001) for individuals aged ≥90 years. Men had a lower risk of impaired nutritional status than women (OR: 0.74; 95% CI: 0.65–0.85; *p* < 0.001). Lower educational attainment (vocational education OR: 1.28, 95% CI: 1.01–1.62, *p* = 0.041; primary education OR: 1.73, 95% CI: 1.37–2.18, *p* < 0.001) was significantly associated with higher risk compared with higher education. Place of residence (urban vs. rural) was not significantly associated with nutritional status. Detailed results are presented in Table 5.

## 4. Discussion

This population-based study demonstrates a clear association between dentition status, denture use, and nutritional status in older adults, confirming that oral health is an important yet frequently overlooked determinant of malnutrition risk. Individuals with edentulism—particularly those without active prosthetic rehabilitation—exhibited poorer MNA-SF scores as well as less favorable laboratory and anthropometric indicators related to frailty, including serum albumin concentration and calf circumference. Serum albumin should therefore be interpreted cautiously as an indicator of nutritional status in older adults, as it may also be influenced by inflammation, chronic disease, and other non-nutritional factors. Similarly, calf circumference primarily reflects muscle mass and risk of sarcopenia rather than nutritional status alone; therefore, it should be interpreted as an indirect indicator related to frailty and nutritional vulnerability. These findings are consistent with previous evidence indicating that impaired oral function contributes to nutritional vulnerability in later life [7,8,9,10].

The observed gradient of worsening nutritional status with increasing tooth loss supports the concept that oral health primarily affects nutrition through functional limitations. It should also be noted that the category of partial dentition (1–19 teeth) represents a heterogeneous group that may include individuals with markedly different chewing abilities. The absence of detailed information on occlusal pairs or functional dentition patterns may therefore partially attenuate the observed associations between dentition status and nutritional outcomes. Tooth loss and inadequate prosthetic rehabilitation reduce masticatory efficiency, leading older adults to avoid foods that require intensive chewing, such as raw vegetables, fruits, nuts, and meat. Consequently, diets may become monotonous, lower in protein and fiber, and deficient in essential micronutrients, increasing the risk of protein–energy malnutrition. These mechanisms are particularly relevant in community-dwelling older adults, where dietary choices are strongly influenced by oral comfort and chewing ability [19,20,21,22].

Beyond mechanical aspects, oral health influences nutritional status through biological and psychosocial pathways. Impaired dentition may also influence bolus preparation and swallowing efficiency. Reduced masticatory performance may lead to inadequate food comminution, which can contribute to swallowing difficulties and increase the risk of dysphagia-related eating problems in older adults. Xerostomia—common in older adults due to polypharmacy and chronic disease—impairs bolus formation and swallowing and further restricts food intake [9,10]. Psychosocial consequences of edentulism, such as reduced self-esteem and avoidance of eating in social settings, may suppress appetite and decrease meal frequency. Functional limitations, social isolation, and cognitive impairment may additionally exacerbate inadequate nutritional intake, creating a bidirectional and self-reinforcing relationship between oral dysfunction and malnutrition [23,24].

An important finding of this study is the observed association between denture use and more favorable nutritional status among edentulous individuals. Denture users were more likely to be well nourished, whereas lack of prosthetic rehabilitation was substantially more common among malnourished participants. These observations are in line with previous studies suggesting that denture use may be associated with better nutritional and functional outcomes in edentulous older adults [25]. However, in the present cross-sectional study, this finding should be interpreted cautiously because denture use may also reflect differences in socioeconomic status, healthcare access, or overall health behaviors. Although denture use does not fully replicate the function of natural dentition, it appears to represent a modifiable factor that may reduce nutritional risk in edentulous older adults.

The results also align with emerging concepts such as “oral frailty,” which emphasize the cumulative impact of tooth loss, reduced oral function, xerostomia, and eating difficulties on systemic health outcomes. Studies have shown that oral frailty is associated with lower MNA scores, sarcopenia, and increased vulnerability to adverse health outcomes [26,27]. The present findings support this framework by demonstrating consistent associations between dentition status, nutritional screening results, and indicators related to frailty.

A major strength of this study is its large, nationally representative sample, which enhances generalizability and allows robust assessment of associations across dentition and nutritional categories. The integration of a validated nutritional screening tool (MNA-SF) with objective measures of oral health and clinically relevant indicators such as albumin concentration and calf circumference represents an additional strength. Importantly, the study provides novel population-based evidence from Central and Eastern Europe, a region where edentulism and unmet prosthetic needs remain highly prevalent but underrepresented in the nutrition literature.

Improvements in dental health observed in recent years among Polish older adults may reflect several factors, including improved oral health awareness, generational changes in preventive dental care, and expanded access to dental services following socioeconomic changes over the past decades. Although certain dental services are publicly funded within the Polish healthcare system, prosthetic rehabilitation often involves out-of-pocket costs, which may contribute to inequalities in oral health and access to dental care.

Several limitations should be acknowledged. First, the cross-sectional design precludes causal inference; while poor oral health may contribute to malnutrition, malnutrition itself may worsen oral function through sarcopenia, impaired wound healing, or mucosal changes. Second, the regression models were adjusted for key sociodemographic variables but did not include detailed measures of comorbidity burden, functional status, or pharmacological treatment. These factors may influence both oral health and nutritional status and may therefore contribute to residual confounding. Third, oral function was not directly assessed using performance-based measures such as masticatory efficiency or tongue strength, and denture quality and fit were not evaluated. These factors may modify the relationship between denture use and nutritional outcomes and should be addressed in future studies. Fourth, although PolSenior2 was designed as a nationally representative survey of older adults in Poland, some degree of selection bias cannot be excluded. As in other population-based studies involving home visits, participation may have been influenced by respondents’ availability, health status, and willingness to participate.

Despite these limitations, the findings have important clinical and public health implications. Incorporating basic oral health assessment—such as dentition status and denture use—into routine nutritional screening may help identify older adults at increased risk of malnutrition. In clinical practice, oral rehabilitation should be considered alongside dietary counselling and, when appropriate, individualized nutritional support or supplementation aimed at maintaining muscle mass, functional status, and metabolic resilience in older adults. Given the potentially beneficial role of prosthetic rehabilitation, improving access to appropriate dental care and dentures may represent a feasible strategy to support nutritional resilience in ageing populations. Future longitudinal studies are needed to clarify causal pathways and to evaluate whether improvements in oral rehabilitation translate into sustained benefits in dietary quality, nutritional status, and health outcomes.

## 5. Conclusions

In a nationally representative sample of Polish older adults, dentition status was significantly associated with nutritional status assessed using the Mini Nutritional Assessment—Short Form. Edentulism was linked to a markedly higher risk of malnutrition or risk of malnutrition compared with functional dentition, while partial dentition showed a weaker and non-significant association.

Among edentulous individuals, denture use appeared to be associated with better nutritional status, whereas lack of active prosthetic rehabilitation was more common in malnourished participants. These findings suggest that prosthetic rehabilitation may represent a potentially important component of strategies aimed at reducing nutritional vulnerability in older adults with complete tooth loss.

The observed associations are likely driven by a combination of reduced masticatory ability, limited dietary diversity, xerostomia, oral discomfort, and psychosocial factors related to tooth loss and social participation. Together, these mechanisms highlight the complex and multidimensional links between oral health and nutrition in later life.

From a clinical and public health perspective, assessment of dentition status and denture use may represent a valuable complement to standard nutritional screening tools in older adults. Integrating basic oral health assessment into routine nutritional screening, together with improved access to prosthetic rehabilitation, dietary counselling, and appropriate nutritional support, may help identify vulnerable individuals earlier and support healthy ageing.

## Figures and Tables

**Table 1:** Characteristics of the study population.

Characteristic	Category	*n*	%
Sex	Women	2690	51.6
	Men	2524	48.4
Age group (years)	60–69	1773	34.0
	70–79	1725	33.1
	80–89	1264	24.2
	≥90	452	8.7
Place of residence	Rural	1885	36.2
	Town with population <50,000	1324	25.4
	City with population of 50,000–200,000	978	18.8
	City with population >200,000	1027	19.7
Education	Primary or less	1522	29.8
	Vocational	1262	24.7
	Secondary/post-secondary	1677	32.8
	Higher	652	12.8
Dentition status	Edentulous (0 teeth)	1900	36.4
	Partial dentition (1–19 teeth)	2508	48.1
	Functional dentition (≥20 teeth)	806	15.5

Data are presented as percentages (%), unless otherwise indicated. Percentages may not sum to 100% due to rounding. Dentition status was classified as functional dentition (≥20 teeth), partial dentition (1–19 teeth), or edentulism (0 teeth).

**Table 2:** Dentition status according to MNA-SF nutritional categories.

Nutritional Status (MNA-SF)	Dentition Status	%	95% CI
Well nourished	Edentulous	26.2	23.8–28.5
	Partial dentition	50.3	48.1–52.4
	Functional dentition	23.6	21.2–25.9
At risk of malnutrition	Edentulous	41.8	38.2–45.4
	Partial dentition	45.3	41.7–48.9
	Functional dentition	12.9	9.5–16.2
Malnourished	Edentulous	46.9	34.9–58.9
	Partial dentition	43.8	33.0–54.6
	Functional dentition	9.3	0.9–17.7

Percentages are calculated within MNA-SF categories. Nutritional status was assessed using the Mini Nutritional Assessment—Short Form (MNA-SF) and classified as well nourished (12–14 points), at risk of malnutrition (8–11 points), or malnourished (0–7 points). CI—confidence interval.

**Table 3:** Dentition status according to albumin concentration and calf circumference.

Albumin Category	Dentition Status	%	95% CI
Albumin <35 g/L	Edentulous	38.0	29.4–46.6
	Partial dentition	46.5	36.2–56.8
	Functional dentition	15.6	6.8–24.4
Albumin ≥35 g/L	Edentulous	29.7	27.5–31.9
	Partial dentition	49.5	47.7–51.3
	Functional dentition	20.8	18.9–22.8
Calf circumference category			
<31 cm	Edentulous	44.1	35.6–52.5
	Partial dentition	47.1	39.7–54.4
	Functional dentition	8.8	5.1–12.5
31–32.99 cm	Edentulous	45.2	38.2–52.2
	Partial dentition	42.2	34.3–50.2
	Functional dentition	12.6	7.0–18.1
≥33 cm	Edentulous	28.3	26.2–30.4
	Partial dentition	49.9	48.1–51.8
	Functional dentition	21.7	19.6–23.9

Hypoalbuminemia was defined as serum albumin concentration <35 g/L. Low calf circumference was defined as <31 cm, indicating increased risk of sarcopenia. Percentages are calculated within categories for albumin concentration and calf circumference. CI—confidence interval.

**Table 4:** Denture use among edentulous participants according to nutritional status.

Nutritional Status (MNA-SF)	Denture Use Status	%	95% CI
Well nourished	No dentures/does not use	7.0	4.9–9.1
	Uses dentures	93.0	90.9–95.1
At risk of malnutrition	No dentures/does not use	15.9	11.8–20.0
	Uses dentures	84.1	80.0–88.2
Malnourished	No dentures/does not use	22.8	12.8–32.9
	Uses dentures	77.2	67.1–87.2

The analysis includes only edentulous participants (0 natural teeth). Denture use refers to current use of complete dentures in the maxilla and/or mandible at the time of examination. Percentages are calculated within MNA-SF nutritional categories. CI—confidence interval.

**Table 5:** Multivariable logistic regression analysis of impaired nutritional status.

Variable	Category	Adjusted OR	95% CI	*p*-Value	Significance Codes
Dentition status	Functional dentition	1.00	Reference	—	
	Partial dentition	1.22	0.98–1.52	0.077	
	Edentulous	1.66	1.32–2.10	<0.001	***
Sex	Women	1.00	Reference	—	
	Men	0.74	0.65–0.85	<0.001	***
Age group	60–69	1.00	Reference	—	
	70–79	1.27	1.07–1.51	0.006	**
	80–89	2.37	1.97–2.84	<0.001	***
	≥90	5.19	4.04–6.65	<0.001	***
Place of residence	Urban	1.00	Reference	—	
	Rural	0.94	0.82–1.09	0.405	
Education	Higher	1.00	Reference	—	
	Secondary	1.04	0.83–1.31	0.723	
	Vocational	1.28	1.01–1.62	0.041	*
	Primary or less	1.73	1.37–2.18	<0.001	***

Impaired nutritional status was defined as MNA-SF <12 (malnutrition or risk of malnutrition). Odds ratios (ORs) with 95% confidence intervals (95% CIs) were estimated using multivariable logistic regression. Functional dentition (≥20 teeth) served as the reference category for dentition status. Models were adjusted for age, sex, education level, and place of residence. CI—confidence interval; OR—odds ratio. Significance codes: * *p* < 0.05; ** *p* < 0.01; *** *p* < 0.001.

## Data Availability

The data presented in this study are available on request from the corresponding author.

## Abbreviations

The following abbreviations are used in this manuscript:

MNA-SF	Mini Nutritional Assessment-Short Form
OR	Odds Ratio
CI	Confidence Interval
